# Mitochondrial Genetic Diversity of *Bemisia tabaci* (Gennadius) (Hemiptera: Aleyrodidae) Associated with Cassava in Lao PDR

**DOI:** 10.3390/insects13100861

**Published:** 2022-09-22

**Authors:** Ana M. Leiva, Khonesavanh Chittarath, Diana Lopez-Alvarez, Pinkham Vongphachanh, Maria Isabel Gomez, Somkhit Sengsay, Xiao-Wei Wang, Rafael Rodriguez, Jonathan Newby, Wilmer J. Cuellar

**Affiliations:** 1 Cassava Program, Crops for Nutrition and Health, International Center for Tropical Agriculture (CIAT), The Americas Hub, Km 17 Recta Cali-Palmira, Cali 763537, Colombia; 2 Plant Protection Center (PPC), Department of Agriculture, Ministry of Agriculture and Forestry, Vientiane P.O. Box 811, Laos; 3 Department of Biological Sciences, Universidad Nacional de Colombia UNAL-Palmira, Palmira 763533, Colombia; 4 Institute of Insect Sciences, Zhejiang University, Hangzhou 310058, China; 5 Cassava Program Asia Office, Crops for Nutrition and Health, International Center for Tropical Agriculture (CIAT), Laos Country Office, Vientiane P.O. Box 783, Laos

**Keywords:** *Bemisia tabaci*, whitefly, nanopore, mtCOI, Southeast Asia, haplotype, Cassava Mosaic Disease

## Abstract

**Simple Summary:**

The whitefly species *Bemisia tabaci* is a known pest of cassava and a vector of cassava geminiviruses in Africa and India, but its role in the recent spread of Cassava Mosaic Disease (CMD) in Southeast Asia is not well known. This is in part due to a lack of data on the occurrence and distribution of *B. tabaci* in this region. We show here the first results of any country-wide survey and identification of *B. tabaci* colonizing cassava in Lao PDR.

**Abstract:**

Cassava Mosaic Disease (CMD) caused by Sri Lankan cassava mosaic virus (SLCMV), has rapidly spread in Southeast Asia (SEA) since 2016. Recently it has been documented in Lao PDR. Previous reports have identified whitefly species of *B. tabaci* as potential vectors of CMD in SEA, but their occurrence and distribution in cassava fields is not well known. We conducted a countrywide survey in Lao PDR for adult whiteflies in cassava fields, and determined the abundance and genetic diversity of the *B. tabaci* species complex using mitochondrial cytochrome oxidase I (mtCOI) sequencing. In order to expedite the process, PCR amplifications were performed directly on whitefly adults without DNA extraction, and mtCOI sequences obtained using nanopore portable-sequencing technology. Low whitefly abundances and two cryptic species of the *B. tabaci* complex, Asia II 1 and Asia II 6, were identified. This is the first work on abundance and genetic identification of whiteflies associated with cassava in Lao PDR. This study indicates currently only a secondary role for Asia II in spreading CMD or as a pest. Routine monitoring and transmission studies on Asia II 6 should be carried out to establish its potential role as a vector of SLCMV in this region.

## 1. Introduction

Cassava (*Manihot esculenta* Crantz) is an important smallholder cash crop and a staple food in the tropics of Latin America, Africa, Asia and Southeast Asia (SEA) [1,2,3]. However, cassava is affected by many diseases, critically including Cassava Mosaic Disease (CMD), caused by single or mixed infections of up to 10 species of viruses belonging to the genus *Begomovirus* in Africa, and two in Asia [4,5]. In 2015, CMD was first reported in SEA in Cambodia and its causal agent was identified as an isolate of Sri Lankan cassava mosaic virus (SLCMV) [6]. Since then, the disease has been reported from Vietnam, China, Thailand and, most recently, it has been documented in Lao PDR, creating a phytosanitary emergency throughout SEA [5,6,7,8,9,10]. Since cassava is a vegetatively propagated crop, infectious diseases can be easily spread through sharing infected planting material (stakes), under limited phytosanitary regulation [11,12] and this could explain the rapid expansion of CMD observed in SEA. Nevertheless, CMD is also a vector-borne disease, transmitted by various species of the *Bemisia tabaci* (Gennadius) whitefly complex (Hemiptera: Aleyrodidae) [11,13].

Whiteflies are vectors for at least 320 species of plant viruses belonging to the genera *Begomovirus*, *Carlavirus*, *Crinivirus*, *Ipomovirus*, *Polerovirus* and *Torradovirus* [14,15,16,17]. Notably, the whitefly species *Bemisia tabaci* is the sole vector of begomoviruses [18,19] and is considered a complex of at least 44 reproductively isolated but morphologically indistinguishable cryptic species [20]. Assigning the status of individual species within the complex classification of *B. tabaci* currently relies on network analysis of its mitochondrial cytochrome oxidase I (mtCOI) gene. This method has been widely used in diversity studies of *B. tabaci* [21,22,23,24,25] and is recommended as a barcoding system for whiteflies [26,27].

In several Asian countries such as Bangladesh China, Nepal, Thailand, Malaysia and Vietnam, different studies using mtCOI have identified *B. tabaci* species diversity and their potential as virus vectors [17,28,29,30,31,32]. The relevance of characterizing *B. tabaci* diversity has increased in SEA due to increasing importance of cassava and the recent introduction of CMD in this region [3,5]. 

Lao PDR is the sixth largest exporter of cassava in the world, with an estimated annual production of 2.3 M tons and USD 116 M in exports [33]. However, there is limited information on genetic diversity of *B. tabaci* populations in Laotian cassava-growing regions and their association with CMD. *B. tabaci* genetic diversity does not change significantly over a farm-scape [34], although it can vary between regions, and regional populations can differ in their ability to transmit begomoviruses. Therefore, the main objectives of this study were to provide the first detailed description of the diversity of the *B. tabaci* complex colonizing cassava in Lao PDR and its potential relation with the recently reported occurrence of CMD in this country [10]. For this, we carried out a country-wide field survey in the main cassava growing regions of Lao PDR and calculated the relative abundance of adult *B. tabaci* per province (10 provinces). To identify the genetic diversity, we sequenced 867 nt of the 3′ half of the mtCOI gene using a rapid and high throughput genotyping tool based on Oxford Nanopore Technologies (ONT). Two indigenous cryptic species, Asia II 1 and Asia II 6, were identified, which were grouped into 14 haplotypes distributed throughout the country. This is the first study on whitefly diversity in Lao PDR. Furthermore, unlike previous studies relying on DNA extraction, PCR amplicon cloning and subsequent sequencing of the mtCOI gene, the current study used more efficient and less cumbersome nanopore sequencing. The methodology employed in this study will simplify future *B. tabaci* genetic diversity studies and monitoring of regional abundance. Such studies will be pivotal in developing effective regional management programs for *B. tabaci*.

## 2. Materials and Methods

### 2.1. Plant Material and Whitefly Sampling

Between June and July 2020, 70 cassava fields were inspected for adult whiteflies (*Bemisia tabaci*) three to six months after planting to calculate the relative abundance of adult whiteflies, in 10 different provinces of Lao PDR (Table 1).

The relative abundance of whiteflies per field was measured as the total number of whiteflies observed in an inspected field divided by the total number of inspected plants within 1 ha. Previous analyses considering the crop density per ha, carried out at CIAT’s research station, indicated that a total of 30 observations was most efficient in terms of minimizing the standard error and time required to collect data per field (Figure S1). To obtain the data by province we used the average number of all the fields surveyed in each province. To count the whiteflies per plant we inspected every fourth cassava plant following a diagonal transect across a 1 ha cassava field (Figure S1) by gently turning and photographing the first fully expanded leaf of one of the branches; this is the preferred leaf for several whitefly species of cassava [35]. The number of adults settled on each leaf was later counted using the photographic record. Additionally, when possible, whitefly adults were collected by aspiration and then immediately stored in 96% ethanol. Due to the low whitefly population observed, whiteflies could only be collected for molecular analysis from 56 fields and 64 individuals were sequenced. The geographic coordinates per field were recorded using a handheld GPS unit, together with the name of the location (province, district, village). Data on CMD incidence in Lao PDR were collected as described in [10] and are stored in the PestDisPlace database [36].

### 2.2. Mitochondrial COI (mtCOI) DNA Amplification

Mitochondrial COI (mtCOI) DNA amplification was carried out by removing the whiteflies from the ethanol, and quickly dropping the insects into tubes containing the PCR reaction mix; there was no need to macerate the samples or to include any additional PCR steps. For amplification we used primers 2195-Bt-F (5′-TGRTTTTTTGGTCATCCRGAAGT-3′) and C012-Bt-sh2-R (5′-TTTACTGCACTTTCTGCC-3′) [37]. These primers amplify 867 bp of the 3′ half of the mtCOI gene. The PCR reaction mix was prepared with 12 μL 2X GoTaq Green Master Mix 2X (Promega Corp^®^), 0.5 μL of each primer (10 pmol) and 7 μL ultrapure water (Invitrogen, Life Technologies, CA, USA) to complete a final volume of 20 μL. PCR was carried out at 95 °C for 5 min (initial denaturation of template DNA), followed by 35 cycles at 94 °C for 40 s, 56 °C for 30 s for annealing, and 72 °C for 45 s for extension, with a final extension at 72 °C for 10 min. The PCR products were run on a 1% agarose gel in 1× TBE buffer stained with GelRedTM (Biotium, Fremont, CA, USA). DNA bands were visualized using a Gel Doc™ XR+ Gel Documentation System (BioRad, CA, USA). As no unspecific bands were observed, libraries for sequencing were prepared directly from the PCR reaction mix.

### 2.3. Nanopore Sequencing

**Figure 1:**
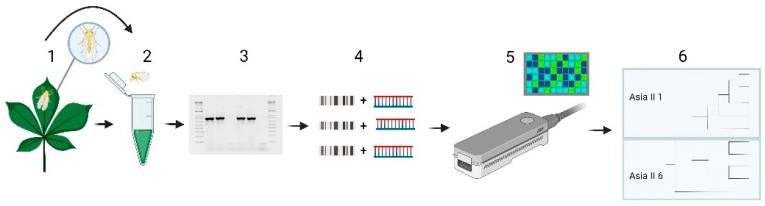
Workflow employed in this work. 1—collected sample, 2—direct PCR using the whole whitefly, 3—confirm the PCR by electrophoresis, 4—library preparation, 5—sequencing using Nanopore Tech, 6—data analysis. Created with Biorender.com (Toronto, ON, Canada).

The sequencing workflow is shown in Figure 1. Briefly, PCR reactions (4 µL each) were diluted with nuclease-free water to obtain 1 µg of total DNA, as determined by the Qubit DNA HS assay (Invitrogen, Life Technologies, CA, USA). Libraries were prepared using the 1D ligation sequencing kit (SQK-LSK109) and native barcodes for genomic DNA (EXP-NBD104 and EXP-NBD114), following manufacturer protocols (ONT). Amplicons were repaired and end-prepped by mixing with 1.75 μL NEBNext FFPE DNA Repair Buffer (New England Biolabs, MA, USA), 1 μL NEBNext FFPE DNA Repair Mix (New England Biolabs, MA, USA), 1.75 μL Ultra II End-prep reaction buffer (New England Biolabs) and 1.5 μL Ultra II End-prep enzyme mix (New England Biolabs). Then, the mixtures were cleaned-up using AMPure XP beads (Beckmann Coulter, CA, USA) as described in the ONT protocol and eluted in 31 μL of nuclease-free water. Adapters were afterwards ligated by mixing 30 μL of amplicons prepared in the previous step, 12.5 μL of ligation buffer (ONT), 5 μL of NEBNext Quick T4 DNA ligase (New England Biolabs) and 2.5 μL of adapter mix (ONT). The amplicons were cleaned-up again using AMPure XP beads and combined to 10 fmol of each sample in one tube. Using 125 μL of short fragment buffer (ONT) as described in the ONT protocol, the pellet was resuspended in 8 μL of elution buffer (ONT). A total volume of 5 μL was used for the sequencing mix, which was then mixed with 10 μL of loading beads (ONT) and 15 μL of sequencing buffer (ONT). We sequenced between 18 and 20 samples per assay, using a Flongle flow cell. The Flongle flow cell was primed with a mix of 117 μL of flush buffer with 3 μL of flush tether, followed by the addition of 30 μL of the sequencing mix. The Flongle was operated using MinKNOW and the total run time was 24 h using a MK1C device (ONT).

### 2.4. Bioinformatic Analysis

Raw FAST5 files pass produced by MK1C device were basecalled under high-accuracy mode using the ONT basecaller Guppy (version 6.0.6; used parameters: guppy_basecaller-input_path PATH-save_path PATH-qscore_filtering-min_qscore 7-flowcell FLO-MIN106-kit SQK-LSK109-cpu_threads_per_caller 4-num_callers 4). The FASTQ files were filtered with a quality Qscore ≥ 9 and only files with this quality were further processed using the next pipeline. The reads were assembled using reference sequences with Minimap2 long-read aligner with 99% accuracy [38]. Then, two different polishers were used for error correction: Pilon, which polishes assemblies created from long-read technologies by overlaying high-quality data to improve local base quality [39], and Medaka v.1.4.4, a neural network-based method to improve accuracy and speed as compared to nanopolish (ONT) [40]. Finally, quality was validated using Qualimap v.2.2.1 [41].

### 2.5. Statistical Analysis

The estimation of nucleotide (π) and haplotype (h) diversities, as well as the number and allocation of haplotypes, was determined using DnaSP 6.12.03 software [42] for sequences generated in this work. Sequence alignments were generated with MUSCLE implemented in Geneious Prime (Biomatters Ltd., Auckland, New Zealand) using 59 sequences generated in this work and 45 reference sequences collected from Genbank. Haplotype networks were determined with Network 10.2.0.0 software (www.fluxus-engineering.com/sharenet.htm (accessed on 31 July 2022)) using the Neighbor-Joining algorithm. On the other hand, to determine phylogenetic relationships, we used fourteen haplotypes from Lao PDR plus 104 sequences obtained from Genbank (https://www.ncbi.nlm.nih.gov/nuccore/?term=Bemisia+tabaci+coi (accessed on 31 July 2022)) representing all whitefly (*Bemisia tabaci*) cryptic species reported so far (Table S1). A phylogenetic tree was inferred applying the Maximum Likelihood optimization criterion using IQ-TREE v 2.0.3 [43] with the model TIM3+I+G4. Support values for each node were calculated based on bootstrapping with 1000 replicates. Finally, the tree was visualized using FigTree v1.4.4 (http://tree.bio.ed.ac.uk/software/figtree/ (accessed on 31 July 2022)). Additionally, to identify whether a population has undergone a recent population expansion event, the mutation frequencies in the sequences were calculated using the tests of Tajima’s D implemented in MEGA-X software [44]. This is determined by the difference between the average number of nucleotide differences and the number of segregating sites estimated from pairwise comparisons [45].

## 3. Results

### 3.1. Whitefly Abundance and CMD Incidence

**Figure 2:**
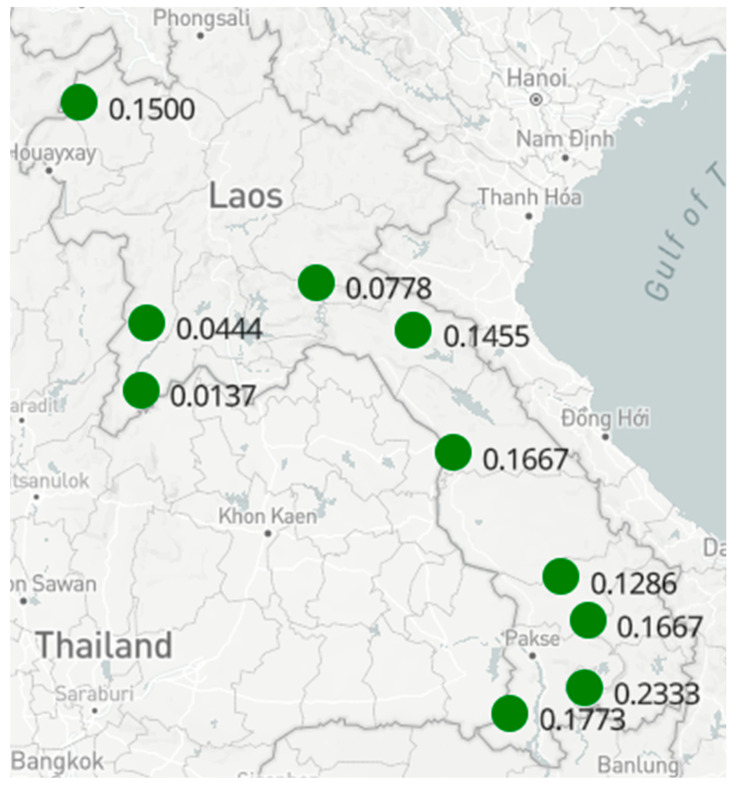
Map of Lao PDR showing abundance of cassava whiteflies per surveyed province. https://pdptest.ciat.cgiar.org/projects/PDP_00088/map?provinces-4.79/15.34/98.13. Accessed on 15 March 2021.

In this study, the number and distribution of collection sites and number of whiteflies collected varied, depending on the area of cassava cultivated in each province and the relative abundance of *B. tabaci* in those fields (Figure 2). The localities with the highest number of fields sampled were Champasack and Borlikhamsai (representing the largest cassava cultivation areas in Lao PDR) (Table 1). In general, whitefly abundances were below 0.23 per leaf, which is 10 times lower than the minimum abundance values observed in cassava fields from Africa (Table 1). This sampling protocol allowed us to determine the relative abundance of whiteflies and the incidence of CMD symptoms at the sampled sites. CMD was only recorded in fields of the southern provinces of Champasack and Attapue, with incidences of 2.4% and 20.3%, respectively (Table S1). At 3–6 months after planting, a distinction is possible between infections derived from infected planting material versus whitefly-derived infections; if symptoms are observed only in the top leaves, this indicates that infection occurred after planting, suggesting whitefly transmission. When the planting material comes from infected plants, one also observes CMD symptoms in the older leaves [46]. 7.6 % of plants showed clear CMD symptoms only in the top young leaves (3–6 months after planting), while in most symptomatic plants, leaf symptoms were distributed along the whole plant. 

### 3.2. Genetic Diversity 

Out of 64 individuals sequenced with the MK1C (ONT) sequencer which had a coverage greater than 1000× (Table S1), 37 sequences (62.72%) corresponded to Asia II 1 while 22 sequences (37.28%) corresponded to Asia II 6. The average nucleotide frequencies found were 23.4% (A), 41.8% (T), 13.9% (C) and 23.4% (G) for 669 nucleotides aligned against available NCBI reference sequences. In total, 73 polymorphic sites (S) were identified, including two singleton sites and 71 parsimony-informative sites, important for classifying haplotypes (Table 2). In general, the haplotype diversity (h) of the entire population from Lao PDR was 0.743 and the nucleotide diversity (π) presented values of 0.04975. Tests for neutrality on the Asia II 1 data subset were significatively negative (Tajima’s D = −2.168 with *p* < 0.05), suggesting a recent population expansion. On the other hand, neutrality tests for Asia II 6 were close to zero (Tajima’s D = −0.644 with *p* > 0.05) and the sequences in this subgroup showed the highest values of average nucleotide differences (k) (Table 2). 

**Figure 3:**
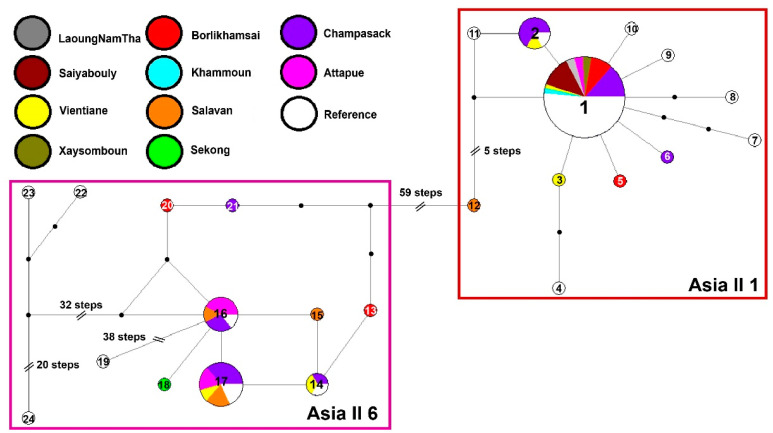
Haplotypes obtained from Median Joining with Network Software. Twenty-four mitochondrial cytochrome oxidase I haplotypes from 104 individuals of Asia II 1 and Asia II 6 are observed. The area of the circles is proportional to haplotype frequency in the dataset. The geographical region of each haplotype is color-coded and the small black circles indicate mutational changes.

When analyzed together with other available COI sequences detected in cassava from Asian countries (n = 45), the network confirmed the presence of haplotypes previously reported in Asia, and revealed a few haplotypes exclusively found in Lao PDR (Figure 3). Asia II 1 sequences were grouped into 12 haplotypes, where the most frequent was H1 (n = 58 sequences). This Asian haplotype was also found in eight provinces from Lao PDR, and it was the only one found in the northern provinces of LaoungNamTha, Saiyabouly, Xaysomboun and Khammoun. 

Asia II 1 haplotypes H3, H5, H12 and H6 were exclusively found in Lao PDR and among these, haplotype H12 (Salavan province) had the highest number of genetic changes. In comparison, Asia II 6 sequences (n = 22) were grouped into eight haplotypes (Table S2) where haplotype H17 was the most frequently found in Lao PDR (four provinces) and where haplotypes H13, H15, H18, H20 and H21 were exclusively found in Lao PDR.

### 3.3. Phylogenetic Analysis

**Figure 4:**
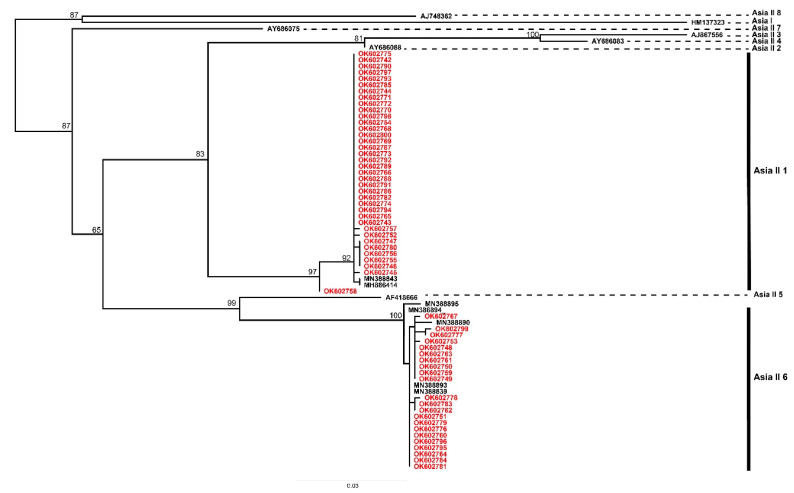
Maximum Likelihood phylogenetic analysis of all consensus sequences from the region. mtCOI using IQTree with 1000 bootstrap replications involving 669 nt of sequences obtained in this study (in red) and reference sequences from whiteflies occurring in Asia. Detailed information on the sequences used in this analysis are included in Table S1.

The phylogenetic reconstruction inferred using Maximum Likelihood (Figure 4) allowed separating and confirming the classification of the whitefly samples into two cryptic species. The Asia II 1 clade comprised 62.7% of sampled whiteflies of this study, and grouped with external references sequences from China, Pakistan, Syria, Thailand and Vietnam (reported in cassava, cotton, mung bean, soybean, sweet potato and tomato). On the other hand, the Asia II 6 clade was represented by 37.3% sequences and eight haplotypes, which were grouped into two clades: H16, H18, H20 and H21 are grouped with a bootstrap support of 51, while H163, H14, H15 and H17 with bootstrap support of 100. Overall, samples from Lao PDR were represented in 14 haplotypes (Figure 3; Table S2), which were included in the phylogenetic analysis, with 262 other sequences from the mtCOI region corresponding to representative samples of different cryptic species of *B. tabaci* (Figure 4). 

## 4. Discussion

In referring to comparative studies on whitefly abundance in cassava fields [49], reports from Africa indicate an average of 100 adults per leaf per plant in Uganda, where severe whitefly-transmitted CMD is endemic [50]. This is in sharp contrast to our observations in Lao PDR (Table 1). There are no other reports on whitefly abundance in the region; however, colleagues in Cambodia and Vietnam report preliminary whitefly abundance data ranging from 0.02 to 0.23 adults/leaf per plant (*not shown*). Interestingly, provinces in Lao PDR with the relative highest abundance of whiteflies correspond to fields located in the southern provinces where CMD also occurs (Table S2) [10]; nevertheless, abundance of adult whiteflies cannot be directly related to incidence of CMD as it can take several weeks for the symptoms to develop after whitefly transmission [46,49,50]. Data was collected 3–6 months after planting as recommended in previous works, a time where populations of whiteflies are most numerous and we can still distinguish between infections propagated by distributing infected planting material versus whitefly-derived infections [46,49]. The results show abundance values significantly lower compared to those in Tanzania and Nigeria, where the superabundance of whiteflies is considered the main factor for the transmission of CMD-causing viruses [50,51,52]. Furthermore, the presence of CMD symptoms in the older leaves of most affected plants suggest at this point a major role for distribution of infected planting material, rather than whiteflies, in the transmission of the disease in Lao PDR. 

At this moment we cannot consider cassava as a major host of Asia II in SEA. In comparison, cassava-infesting *B. tabaci* in sub-Saharan Africa (SSA) is rarely found on other hosts under field conditions, unlike other non-cassava *B. tabaci* in Africa such as MED, Indian Ocean and MEAM1, which do not colonize cassava under field conditions [53]. In contrast, Asia II has been reported in other crops such as cotton and several solanaceous hosts in Asia (see below). Higher whitefly abundances in other crops near cassava fields suggest that cassava is not the main host of Asia II, implying a host effect on the low abundance of *B. tabaci* in cassava in SEA [53].

Direct PCR amplification of the mtCOI region, without DNA extraction (Figure 1), produced clear bands and no PCR inhibition was observed We obtained the expected amplification products (867 bp) from all the samples collected in the different cassava fields and no unspecific band was observed. Sequences obtained using ONT Flongle cells had high levels of coverage, confirming the efficiency of this protocol for the rapid, effective and low-cost sequencing of fragments derived from PCR [54,55]. The protocol described here allowed for the rapid sequencing and bioinformatic analysis of barcoded samples in a single run, offering a more efficient alternative to Sanger sequencing [56]. Although the main problem with nanopore sequencing is its still relatively high per-base error rate in raw reads compared to other sequencing techniques, these are offset by the high coverages obtained and good bioinformatics handling [56]. Previous reports indicate that a 100× coverage is adequate to establish an accurate genotype; furthermore, advances in nanopore sequencing technology, in combination with a dedicated data analysis pipeline and good coverage, have proven to be comparable in performance to conventional Sanger sequencing applied to PCR amplicons, as is the case in this work [57,58].

Cryptic whitefly species Asia II 1 and Asia II 6 have been previously identified as indigenous species in cassava fields in southern Vietnam, Thailand and China [29,59]. The haplotype network reconstructed on the analysis of 14 haplotypes from Lao PDR and 15 haplotypes from various regions of the world clearly showed differences among the indigenous cryptic species of *B. tabaci* Asia II 1 and Asia II 6 (Figure 3), in agreement with the phylogenetic analysis results (Figure 4). The difference between the two cryptic species is given by 59 mutational changes according to the network of haplotypes. A similar range has been observed between Asia II 1 and Asia I in cotton crops in India, where 57 mutations were detected between the two cryptic species [60]. In addition, they show a sequence divergence greater than 3.5%, a value assigned for the separation of cryptic species in *B. tabaci* [25]. Species status of Asia II 1 and Asia II 6 is also supported by previous studies, where mating crosses have shown evidence of reproductive incompatibilities [61,62,63]. 

Asia II 1 was the most frequent species found in the cassava fields, and is the one found also as the dominant species in cotton in Pakistan and in soybean in India [64,65] Although Asia II 1 was more dominant in Lao PDR, Asia II 6 showed a higher haplotype diversity (0.88). Interestingly, Asia II 6 in China forms at least two separate networks, suggesting a distinct evolutionary history for this species in that country [22]. Network analysis does not involve many of the assumptions of phylogenetic reconstruction methods, such as the existence of an ancestral sequence or branching relationships [66]; therefore, haplotype network analysis is useful as an empirical prediction of boundaries within a species complex [67,68,69]. The neutrality test was negative for both species, indicating that their expansion was limited within the local area. Meanwhile the positive value of the Tajima’s D statistics with total population indicated balanced selection [45].

Asia II 1 is an efficient vector of SLCMV, in comparison to MED and MEAM species [32], and is also a vector for Multan cotton leaf curl virus, a pathogen causing significant yield losses in cotton [70]. The phytosanitary importance of Asia II 1 is further highlighted by its recorded resistance to insecticides [71,72]. On the other hand, the Asia II 6 cryptic species occurring in beans, cassava, chili pepper, cotton, cucumber and sweet potato, from China, India, Malaysia, Japan, Thailand and Vietnam [20,29,30,73], as well as in cassava (*this work*), has not yet been identified as a vector of SLCMV. Furthermore, a study in Japan found this species to be sensitive to the majority of the tested pesticides [74]. 

## 5. Conclusions 

Our study presents the first report on the genetic diversity of *B. tabaci* whitefly populations collected in Lao PDR, at the haplotype level (fourteen groups of *B. tabaci* haplotypes). The two whitefly cryptic species (Asia II 1 and Asia II 6) are found throughout the country, including the southern provinces where CMD has been reported. Cryptic species Asia II 1, a known vector of SLCMV, are widespread in the region; however, in Lao PDR, low abundances of whiteflies and the distribution of CMD symptoms in infected plants suggest, at this time, a secondary role for Asia II in the dissemination of CMD or as a pest, as compared to CMD transmission by infected planting material. However, this situation will likely evolve in the near future. Haplotypes unique to Lao PDR have been identified and are useful in studying and tracking the spread of whiteflies at a regional level in parallel with CMD. Further transmission studies with Asia II 6 should be carried out to establish its potential role as a vector of SLCMV and routine monitoring is recommended for timely detection of an increase in abundance of whitefly populations in Lao PDR.

## Figures and Tables

**Table 1:** List of surveyed provinces and abundance data for whiteflies associated to cassava in Lao PDR. Comparative whitefly abundance data from Tanzania and Nigeria is included. SD = standard deviation. The asterisks indicate provinces where CMD was observed. Asia II 1 and its haplotypes are indicated in red.

Country	Province	No. of Fields	Abundance (Per Leaf)	SD	Cryptic Species	Haplotype	Ref.
Lao PDR	LaoungNamTha	2	0.150	0.012	Asia II 1	H1	This work
	Saiyabouly	9	0.041	0.021	Asia II 1	H1	
	Vientiane	5	0.014	0.012	Asia II 1/Asia II 6	H1, H2, H3, H14, H17	
	Xaysomboun	3	0.078	0.040	Asia II 1	H1	
	Borlikhamsai	11	0.145	0.034	Asia II 1/Asia II 6	H1, H5, H20, H13	
	Khammoun	1	0.166	0.000	Asia II 1	H1	
	Salavan	7	0.129	0.031	Asia II 1/Asia II 6	H12, H17, H15, H16	
	Sekong	1	0.167	0.000	Asia II 6	H18	
	Champasack *	25	0.177	0.043	Asia II 1/Asia II 6	H1, H2, H6, H17, H16, H14, H21	
	Attapue *	6	0.233	0.072	Asia II 1/Asia II 6	H1, H17, H16	
Tanzania			2.35–71.99 (14.3)	0.86–22.07	SSA1-SG1-SG2-SG3	ND	[47]
Nigeria		24	2.34–265.5		SSA1-SG5, SSA3, SSA1-SG1-Bemisia afer, MED-ASL	ND	[48]

**Table 2:** Distribution and frequency of cryptic species found in Lao PDR. n = number of sequences; S = number of polymorphic sites; NS = Singleton’s number; Pi = parsimony-informative sites; k = average number of nucleotide differences; h = haplotypic diversity; π = nucleotide diversity; Tajima’s D = neutrality test.

Cryptic Species	n	S	NS	Pi	K	H	π	Tajima’s D
Asia II 1	37	10	9	4	0.727	0.417	0.00109	−2.168
Asia II 6	22	6	2	1	1.433	0.775	0.00214	−0.644
Total	59	73	2	71	33.281	0.743	0.04975	3.923

## Data Availability

The data presented is available in the supplementary tables included with this work.

## Supplementary Materials

The following supporting information can be downloaded at: https://www.mdpi.com/article/10.3390/insects13100861/s1, Figure S1: Field survey protocol; Table S1: Survey data; Table S2: List of COI sequences used.
