# Supplementary material for: Mitochondrial Genetic Diversity of Bemisia tabaci (Gennadius) (Hemiptera: Aleyrodidae) Associated with Cassava in Lao PDR
Source: Insects. 2022 Sep 22;13(10):861. doi: 10.3390/insects13100861 (PMC9604212; doi:10.3390/insects13100861)
Supplement: Supplementary file 1 [file insects-13-00861-s001.zip › Figure S1-Field_survey_protocol-REV.pptx]

## Slide 1
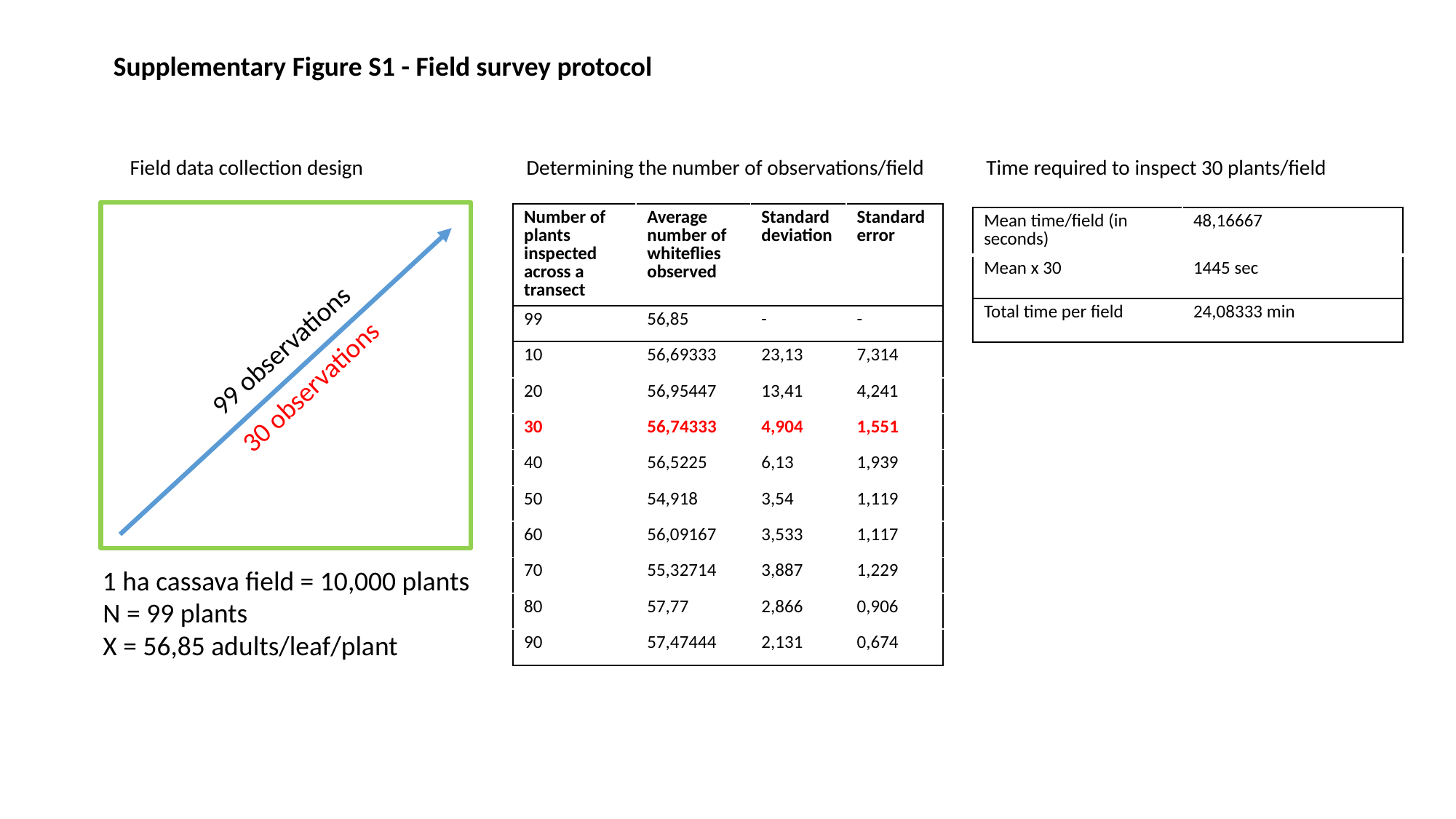

Supplementary Figure S1 - Field survey protocol
Time required to inspect 30 plants/field
Field data collection design
Determining the number of observations/field
| Number of plants inspected across a transect | Average number of whiteflies observed | Standard deviation | Standard error |
| --- | --- | --- | --- |
| 99 | 56,85 | - | - |
| 10 | 56,69333 | 23,13 | 7,314 |
| 20 | 56,95447 | 13,41 | 4,241 |
| 30 | 56,74333 | 4,904 | 1,551 |
| 40 | 56,5225 | 6,13 | 1,939 |
| 50 | 54,918 | 3,54 | 1,119 |
| 60 | 56,09167 | 3,533 | 1,117 |
| 70 | 55,32714 | 3,887 | 1,229 |
| 80 | 57,77 | 2,866 | 0,906 |
| 90 | 57,47444 | 2,131 | 0,674 |
| Mean time/field (in seconds) | 48,16667 |
| --- | --- |
| Mean x 30 | 1445 sec |
| Total time per field | 24,08333 min |
99 observations
30 observations
1 ha cassava field = 10,000 plants
N = 99 plants
X = 56,85 adults/leaf/plant
